# Behaviors and Attitudes Associated With Low Drinking Water Intake Among US Adults, Food Attitudes and Behaviors Survey, 2007

**DOI:** 10.5888/pcd10.120248

**Published:** 2013-04-11

**Authors:** Alyson B. Goodman, Heidi M. Blanck, Bettylou Sherry, Sohyun Park, Linda Nebeling, Amy L. Yaroch

**Affiliations:** Author Affiliations: Heidi M. Blanck, Bettylou Sherry, Sohyun Park, Centers for Disease Control and Prevention, Atlanta, Georgia; Linda Nebeling, National Cancer Institute, Washington, DC; Amy L. Yaroch, Swanson Center for Nutrition, Omaha, Nebraska.

## Abstract

**Introduction:**

Water is vital for life, and plain water is a calorie-free option for hydration. Increasing consumption of drinking water is a strategy to reduce energy intake and lose or maintain weight; however, information on the characteristics of consumers who drink water is limited. Our objective was to describe the characteristics of people who have a low intake of drinking water and to determine associations between their behaviors and attitudes and their intake of water.

**Methods:**

We analyzed data from a nationally representative sample of 3,397 US adults who participated in the National Cancer Institute’s 2007 Food Attitudes and Behaviors Survey. Multivariable logistic regression was used to identify sociodemographic characteristics and health-related behaviors and attitudes associated with self-reported drinking water intake of less than 4 cups per day.

**Results:**

Overall, 7% of adults reported no daily consumption of drinking water, 36% reported drinking 1 to 3 cups, 35% reported drinking 4 to 7 cups, and 22% reported drinking 8 cups or more. The likelihood of drinking less than 4 cups of water daily was significantly higher among participants aged 55 years or older than among those aged 18 to 34 (adjusted odds ratio [AOR], 1.3), among residents of the Northeast than among residents of the South (AOR, 1.4), among participants who consumed 1 cup or less of fruits or vegetables per day than among those who consumed 4.5 cups or more (AOR, 3.0), among participants who did not exercise than among those who exercised 150 minutes or more per week (AOR, 1.7), and among participants who were neither trying to gain nor lose weight than among those trying to lose weight (AOR, 1.3).

**Conclusion:**

Low drinking water intake was associated with age, region of residence, and several unhealthful behaviors and attitudes. Understanding characteristics associated with low drinking water intake may help to identify populations that could benefit from interventions to help adults drink more water.

## Introduction

Adequate water intake has health benefits and is essential for preventing dehydration; dehydration is associated with adverse health effects such as headache, urolithiasis, and impaired cognition (1). Health risks (eg, dental caries, obesity) associated with intake of high levels of calorically sweetened beverages (eg, regular soda, fruit drinks, sports drinks) decrease when plain drinking water is substituted for these beverages (1,2). Water consumption before meals and the replacement of calorically sweetened beverages with water are associated with lower energy intake, and increased plain water intake among adults is associated with significant weight maintenance or loss (3–9). The Dietary Guidelines for Americans 2010 encourages adults to drink water as a healthful means of hydration, and public health organizations and others are bringing this message to communities (10–13).

According to 2005–2008 National Health and Nutrition Examination Survey (NHANES) data, mean plain water intake among US adults (aged ≥20 years) was 4.4 cups for men and 4.3 cups for women (14). Little research has been conducted on the association of individual water consumption practices with diet and meal patterns (15). Although water intake has been associated with individual factors (eg, physical activity, age), little is known about how water intake is related to other food- and health-related behaviors and attitudes (14–17). A comprehensive understanding of how these factors are related to water intake may help identify populations and associated attitudes and behaviors amenable to intervention. For example, clinical or public health messages about water intake could be bundled with messages about associated health behaviors. The purposes of our study were to use a data set with varied information on behaviors and attitudes to quantify daily drinking water intake, to identify sociodemographic and health characteristics associated with low water intake, and to describe the association of food- and health-related behaviors and attitudes with low drinking water intake.

## Methods

We used data for this cross-sectional study from the National Cancer Institute’s Food Attitudes and Behaviors (FAB) Survey, a mail-panel survey of US adults conducted from October through December 2007. The FAB Survey was approved by the National Cancer Institute’s institutional review board (18).

### Participants/recruitment

FAB participants were US residents aged 18 years or older recruited via quota sampling of households in Synovate’s Consumer Opinion Panel (N = 450,000) in the fall of 2006. The FAB survey was first mailed to a stratified random sample of 5,803 adults; 200 additional surveys were mailed later (sample balanced to reflect the US population by region of residence, annual household income, population density, age, and household size); African Americans were oversampled (unweighted response rate, 28%). In total, 3,418 of 6,003 questionnaires were returned (response rate, 57%). For our study, we excluded 167 questionnaires (21 overall incomplete, 23 missing the outcome variable, and 123 missing sociodemographic data), leaving a final study sample of 3,251 respondents. Drinking water intake did not significantly differ between included and excluded respondents. The percentage of respondents with unknown values or missing data for individual exposure variables ranged from less than 0.1% to 7.0%; we excluded respondents with data missing for a given variable from analyses involving that variable.

### Variables

Daily water intake was determined on the basis of participants’ responses to the question, “On average, how many cups of bottled or tap water do you drink each day?” Response options were “none,” “1-3 cups,” “4-7 cups,” and “8 or more cups.” For multivariable logistic regression models, we dichotomized water intake as “less than 4” or “4 or more cups” per day based on the mean water intake (approximately 4.3 cups) among US adults according to 2005–2008 NHANES data (14,15).

Sociodemographic variables were age (18–34 years, 35–54 years, or ≥55 years), sex, race/ethnicity (non-Hispanic white, non-Hispanic black, or other [including Hispanic, non-Hispanic Asian, American Indian, Native Hawaiian, and mixed race]), region of residence (Northeast, Midwest, South, or West), education level (less than a high school diploma, high school degree, some college, or college degree), and annual household income (<$20,000; $20,000-$44,999; $45,000-$74,999; or ≥$75,000).

Variables describing health- and eating-related characteristics for our primary multivariable model were chosen on the basis of likely or known associations with health status and thought to be associated with water intake: weight status based on body mass index (BMI), calculated from self-reported height and weight (underweight/normal weight [BMI <25.0 kg/m^2^], overweight [BMI 25.0 to <30 kg/m^2^], obese [BMI ≥30 kg/m^2^]) (19); daily fruit and vegetable intake (from a validated 16-item screener [18]), categorized as ≤1, >1 to <4.5, or ≥4.5 cups based on recommendations for a 2,000 kilocalorie diet (11); minutes of moderate physical activity per week (0, 1 to <150, ≥150) based on the US Physical Activity Guidelines for adults to get at least 150 minutes of moderate-intensity exercise per week (20); cigarette smoking status (never, former, current); intentions for weight management (neither trying to lose nor gain weight, trying to gain weight, trying to lose weight); hours of television watched on an average day (≤2, >2 to <4, ≥4); and number of hours of sleep on an average night (<6, 6 to <8, ≥8).

We conducted secondary analyses to determine whether the following variables with hypothesized associations with health were related to drinking water intake (while maintaining the parsimony of our multivariate model): how often fruits and vegetables were eaten while growing up (rarely, more than once per week, once daily, more than once daily), whether the primary grocery shopper shops at farmers markets or cooperatives (yes, no), meals eaten per week while watching television (none, 1–4, ≥5 meals), fast food intake (none, once/week, more than once/week), meals per week eaten at the table with family or friends (none, 1–4, ≥5), cups of daily 100% juice intake (none, 1, ≥2 cups), and respondents’ attitudes about “how often worrying about your health has led you to change the way you ate in the past year” (not at all/a little, somewhat, quite a bit/a lot). Variables based on respondents’ agreement with the following: “What I eat doesn’t really affect my health,” “I don’t eat fruits and vegetables as much as I like to because they cost too much,” “It’s hard for me to purchase fruits and vegetables in my neighborhood,” and “I think meals should include some meat” (disagree, neither disagree nor agree, agree).

### Statistical analyses

Data on sex, race/ethnicity, age, education, and annual household income were weighted using 2000 US Census data to create a sample distribution similar to the national distribution. Chi-square tests were used to evaluate the frequency of drinking water intake by exposure variables; significance was set at *P* < .05. Odds ratios (OR) and 95% confidence intervals (CIs) for variables associated with low water intake (<4 cups daily) were calculated from multivariable logistic regression models. The model we used in our primary analysis consisted of sociodemographic variables and selected health- and eating-related variables. In our secondary analyses, we created separate logistic regression models for each exposure variable that were adjusted for age, sex, race/ethnicity, region of residence, income, and education. All analyses were conducted using SAS version 9.2 (SAS Institute, Inc, Cary, North Carolina).

## Results

Of the 3,251 respondents, 43.7% drank less than 4 cups of water per day (Table 1). Approximately 7% reported consuming no drinking water daily, 36% drank 1 to 3 cups, 35% drank 4 to 7 cups, and 22% drank 8 cups or more. On the basis of χ^2^ tests, daily drinking water intake (<4 vs ≥4 cups) varied significantly by age, race/ethnicity, education level, annual household income, weight status, moderate physical activity, fruit and vegetable intake, smoking status, intentions for weight management, and hours of television watched daily.

**Table 1:** Respondents’ Characteristics, Reported Daily Drinking Water Intake, and Adjusted Odds of Drinking Less Than 4 Cups Daily, Food Attitudes and Behaviors Survey, United States, 2007

Characteristic	All, %	Daily Drinking Water Intake		*P* Value^a^	AOR (95%CI) of Drinking <4 Cups Water Daily^b^

		<4 Cups, %	≥4 Cups, %		
Total respondents (n = 3,251)	100.0	43.7	56.3	<.01	NA
Age, y (n = 3,251)					
18–34	31.3	42.9	57.1	.04	1 [Reference]
35–54	35.9	41.4	58.6		0.9 (0.7–1.1)
≥55	32.8	47.0	53.0		1.3 (1.1–1.7)
Sex (n = 3,251)					
Male	47.4	45.1	55.9	.18	1 [Reference]
Female	52.6	42.5	57.5		1.0 (0.8–1.2)
Race/ethnicity (n = 3,251)					
Non-Hispanic white	77.7	44.3	55.7	<.01	1 [Reference]
Non-Hispanic black	11.6	47.7	52.3		0.9 (0.7–1.2)
Other^c^	10.7	34.9	65.1		0.6 (0.5–0.9)
Region (n = 3,251)					
Northeast	19.1	47.0	53.0	.06	1.4 (1.1–1.8)
Midwest	22.5	45.5	54.5		1.1 (0.9–1.5)
South	38.0	43.4	56.6		1 [Reference]
West	20.4	39.3	60.7		1.0 (0.7–1.2)
Highest education level (n = 3,251)					
Less than a high school diploma	13.8	50.2	49.8	<.01	1.1 (0.8–1.5)
High school diploma	31.1	45.5	54.5		1.0 (0.8–1.4)
Some college	29.2	42.6	57.4		1.1 (0.9–1.4)
College degree	25.9	39.4	60.6		1 [Reference]
Annual household income, $ (n = 3,251)					
<20,000	20.6	48.9	51.1	.04	1.2 (0.9–1.6)
20,000–44,999	31.0	43.4	56.6		1.0 (0.8–1.3)
45,000–74,999	22.3	42.1	57.9		1.0 (0.8–1.3)
≥75,000	26.1	41.3	58.7		1 [Reference]
Weight status (BMI, kg/m^2^) (n = 3,111)					
Underweight/normal weight (<25)	32.3	46.7	53.3	<.01	1 [Reference]
Overweight (25-<30)	34.1	43.7	56.3		0.9 (0.7–1.2)
Obese (≥30)	33.6	40.6	59.4		0.8 (0.6–1.0)
Intentions for weight management (n = 3,241)					
Trying to lose weight	57.2	39.6	60.4	<.01	1 [Reference]
Neither trying to lose nor gain	39.4	48.9	51.1		1.3 (1.0–1.6)
Trying to gain weight	3.4	55.3	44.7		1.7 (1.1–2.8)
Moderate physical activity, min/wk (n = 3,119)					
0	31.2	53.3	46.7	<.01	1.7 (1.4–2.1)
1-<150	21.4	45.4	54.6		1.5 (1.2–1.9)
≥150	47.4	37.9	62.1		1 [Reference]
Fruit and vegetable intake, cups/d (n = 3,201)					
≤1	24.3	59.4	40.6	<.01	3.0 (2.2–4.0)
>1-<4.5	57.8	41.4	58.6		1.6 (1.2–2.0)
≥4.5	17.9	30.2	69.8		1 [Reference]
Cigarette smoking status (n = 3,187)					
Never	50.1	42.4	57.6	<.01	1 [Reference]
Former	24.9	39.1	60.9		0.7 (0.6–0.9)
Current	25.0	51.2	48.8		1.1 (0.9–1.4)
Hours of television watched on average day (n = 3,160)					
≤2	43.5	41.8	58.2	<.01	1 [Reference]
>2 -<4	31.6	41.5	58.5		0.9 (0.8–1.1)
≥4	24.9	48.8	51.2		1.2 (1.0–1.6)
Hours of sleep on an average night (n = 3,022)					
<6	13.8	44.6	55.4	.91	1.1 (0.8–1.4)
6 to <8	56.9	43.4	56.6		1 [Reference]
≥8	29.3	43.5	56.5		0.9 (0.8–1.1)

Abbreviations: AOR, adjusted odds ratio; CI, confidence interval; NA, not applicable; BMI, body mass index.

a Chi-square tests were used for each variable to examine differences across categories.

b The final logistic regression model included variables in 1 model to adjust for possible confounders and included a sample of 2,635 adults with complete data for all variables.

c Of the total study sample (3,251), 5% were Hispanic, 2% Asian, 3% American Indian/Native Hawaiian, and 1% mixed race.

Multivariable logistic regression indicated that the likelihood of low drinking water intake (<4 cups/d) was significantly higher among people aged 55 or older (vs aged 18–34), living in the Northeast (vs South), trying to gain weight (vs trying to lose weight), participating in no moderate physical activity and 1 to fewer than 150 minutes per week (vs ≥150 minutes/week), and eating less than 4.5 cups of fruits and vegetables daily (vs ≥4.5 cups/d) (Table 1). Lower odds of drinking less than 4 cups of water per day were observed among respondents of “other” race/ethnicity than among whites and among former smokers than among never smokers.

Results of secondary analyses indicated that drinking water intake differed significantly across many eating-related behaviors (χ^2^ test, *P* < .05) (Table 2). Adjusted ORs indicate that variables significantly related to greater odds for low drinking water intake were recalling eating fruits once daily or less often while growing up (vs more than once daily), recalling eating vegetables once daily or less often while growing up (vs more than once daily), eating fast food more than once per week (vs none), and eating fewer than 5 dinners per week around a table with family or friends (vs ≥5 dinners/week). Shopping at farmers markets or cooperatives (vs not) and intake of 1 or more cups per day of 100% juice (vs none) were significantly related to lower odds for low drinking water intake (Table 2).

**Table 2:** Eating-Related Behaviors, Reported Daily Drinking Water Intake, and Adjusted Odds of Drinking Less Than 4 Cups Daily, by Respondents’ Preferences, Food Attitudes and Behaviors Survey, United States, 2007

Eating-Related Behavior	All, %	Daily Drinking Water Intake			Adjusted OR (95% CI) of Drinking <4 Cups Daily^b^

		<4 cups, %	≥4 cups, %	*P* Value^a^	
How often fruit eaten while growing up (n = 3,215)					
Rarely	11.5	52.3	47.7	<.01	1.8 (1.4–2.4)
More than once/week	36.0	48.3	51.7		1.6 (1.3–2.0)
Once daily	31.2	40.3	59.7		1.2 (1.0–1.5)
More than once daily	21.3	36.1	63.9		1 [Reference]
How often vegetables eaten while growing up (n = 3,226)					
Rarely	4.7	49.6	50.4	<.01	1.6 (1.1–2.4)
More than once/week	20.0	53.1	46.9		1.9 (1.5–2.4)
Once daily	43.3	43.5	56.5		1.3 (1.1–1.6)
More than once daily	32.0	37.3	62.7		1 [Reference]
Primary shopper shops at farmers market/cooperative (n = 3,246)					
No	80.6	45.9	54.0	<.01	1 [Reference]
Yes	19.4	34.2	65.8		0.6 (0.5–0.7)
How often meals eaten while watching TV (n = 3,196)					
None	17.5	41.7	58.3	.41	
1-4 meals/week	33.3	42.6	57.4		1.1 (0.8–1.3)
≥5 meals/week	49.2	44.8	55.2		1.1 (0.9–1.4)
Fast Food Intake (n = 3,176)					
None	29.2	39.2	60.8	<.01	1 [Reference]
Once/week	32.6	40.7	59.3		1.1 (0.9–1.4)
More than once/week	38.2	49.4	50.6		1.6 (1.3–2.0)
Dinners eaten around a table with family or friends (n = 3,145)					
None	20.9	47.5	52.5	<.01	1.3 (1.1–1.7)
1-4 times/week	41.2	45.1	54.9		1.3 (1.1–1.5)
≥5 times/week	37.9	39.1	60.9		1 [Reference]
Daily intake of 100% juice, cups (n = 3,238)					
0	21.6	51.3	48.7	<.01	1 [Reference]
1	62.7	43.8	56.2		0.8 (0.6–0.9)
≥2	15.7	33.3	66.7		0.5 (0.4–0.6)

Abbreviations: OR, odds ratio; CI, confidence interval.

a Chi-square tests were used for each variable to examine differences across categories.

b Multivariable logistic regression model included exposure variable and age, sex, race/ethnicity, education, income and region.

Greater odds of low drinking water intake were significantly related to various attitudes/beliefs about food and health (Table 3). Factors associated with higher odds for low drinking water intake included replying to survey questions as follows: “agree” or “neither disagree nor agree” that “what I eat doesn’t really affect my health” (vs “disagree”); “not at all/a little” or “somewhat” to “How often has worrying about your health led you to change what you ate in the past year?” (vs “quite a bit/a lot”); “agree” or “neither disagree nor agree” that “I don’t eat fruits and vegetables as much as I like to because they cost too much” (vs “disagree”); “agree” that “It’s hard for me to purchase fruits and vegetables in my neighborhood” (vs “disagree”); and “agree” that “I think meals should include some meat” (vs “disagree”).

**Table 3:** Attitudes and Beliefs Related to Eating and Health, Reported Daily Drinking Water Intake, and Adjusted Odds Ratios of Drinking Less Than 4 Cups Daily, by Respondents’ Attitudes, Food Attitudes and Behaviors Survey, United States, 2007

Eating-Related Behavior	All, %	Daily Drinking Water Intake			Adjusted OR (95% CI) of drinking <4 cups daily^b^

		<4 cups, %	≥4 cups, %	*P* Value^a^	
What I eat doesn’t really affect my health (n = 3,189)					
Disagree	71.6	41.5	58.5	<.01	1 [Reference]
Neither disagree nor agree	15.6	49.1	50.9		1.3 (1.0–1.6)
Agree	12.8	48.8	51.2		1.2 (1.0–1.6)
How often has worrying about your health led you to change what you ate in the past year? (n = 3,236)					
Not at all/a little	46.3	47.2	52.8	<.01	1.7 (1.4–2.0)
Somewhat	31.5	44.4	55.6		1.5 (1.2–1.9)
Quite a bit/a lot	22.2	35.5	64.5		1 [Reference]
I don’t eat fruits and vegetables as much as I like to because they cost too much (n = 3,211)					
Disagree	45.9	40.3	59.7	<.01	1 [Reference]
Neither disagree nor agree	23.7	46.7	53.3		1.3 (1.1–1.6)
Agree	30.3	46.9	53.1		1.3 (1.1–1.6)
It’s hard for me to purchase fruits and vegetables in my neighborhood (n = 3,205)					
Disagree	76.8	42.8	57.2	.15	1 [Reference]
Neither disagree nor agree	12.1	44.6	55.4		1.0 (0.8–1.3)
Agree	11.1	48.9	51.1		1.3 (1.0–1.6)
I think meals should include some meat (n = 3,189)					
Disagree	15.9	37.8	62.2	<.01	1 [Reference]
Neither disagree nor agree	22.5	38.0	62.0		1.0 (0.8–1.3)
Agree	61.6	47.3	52.7		1.5 (1.2–1.8)

Abbreviations: OR, odds ratio; CI, confidence interval.

a Chi-square tests were used for each variable to examine differences across categories.

b The multivariable logistic regression model included 1 exposure variable and age, sex, race/ethnicity, education, annual household income, and region.

## Discussion

Our findings indicated that nearly half of respondents drank less than 4 cups per day of water (ie, bottled or tap water) and that 56% of respondents reported drinking 4 or more cups of water daily. These results are consistent with those based on 2005–2008 NHANES data, which indicated that US adults consumed an average of 4.3 cups of water per day (14,15). The biologic requirement for water may be met with plain water or via foods and other beverages. Results from previous epidemiologic studies indicate that water intake may be inversely related to volume of calorically sweetened beverages and other fluid intake (4).

Our results indicated that low drinking water intake was associated with many demographic characteristics, including older age. Despite being susceptible to dehydration due to increased prevalence of chronic diseases and the use of multiple medications, older adults have lower fluid consumption primarily due to a decrease in thirst (1,21). Previous studies indicate that water consumption decreases with age; a study of 4,112 US adults by Kant et al found lower plain water intake among older US adults (15,21,22). Kant et al reported no significant differences in water intake by race/ethnicity (15), whereas we found significantly higher intake among respondents in the “other” race/ethnicity category than among whites. The reasons for this association are unclear (FAB was not powered to detect differences among subgroups in this diverse category). In a study of 4,292 Florida students in grades 6 through 8, Park et al found significantly lower odds of low drinking water intake among Hispanic/Latino or “other”/non-Hispanic adolescents than among white adolescents (adjusted OR = 0.79 and OR = 0.76, respectively), results that are similar to those we obtained among adults (23). Although our study found no association between drinking water intake and education or household income in multivariable models, previous studies reported that plain water intake is positively associated with years of education but not associated with poverty-income ratio (15). An analysis of the US Department of Agriculture Nationwide Food Consumption Survey of 1977 found lower tap water intake in the Northeast (1.2 L/d) than in other regions (1.4 L/d), possibly due to greater need for water among residents in regions with warm or humid climates (24).

Our findings of associations between water intake and certain behaviors were similar to those found in previous research. Meeting the national recommendation for 150 minutes per week of moderate physical activity was associated with significantly higher drinking water intake in this and a previous study (15), which is not surprising given that physical activity leads to increased hydration needs due to sweating (1). The results of our multivariable regression analysis showed no association between water intake and time spent watching television, which is consistent with results of a study among 3,867 US children and adolescents (25). Our finding that former smokers were likely to drink more water than those who never smoked might be explained by the common practice of encouraging participants in tobacco cessation programs to increase their water intake (26).

Low fruit and vegetable intake, which epidemiologic studies link to higher risk of chronic disease (11), was associated with drinking significantly less water in multivariable regression models. In addition, in models controlled for sociodemographic variables, respondents with unhealthful eating behaviors and attitudes (eg, high fast-food intake) drank significantly less water, whereas healthful eating behaviors and attitudes (eg, shopping at farmers markets) were related to drinking more water. These results, which are consistent with findings from previous epidemiologic studies (14–17), add to a growing body of evidence that drinking water intake is associated with healthful dietary practices and attitudes. Whether drinking water supports these healthful dietary patterns or simply coexists with them is unclear. Nonetheless, this evidence suggests that health educators or health care practitioners aiming to promote increased water intake should keep in mind that low water consumption may be closely tied to other unhealthful behaviors.

In our study, respondents trying to lose weight consumed significantly more water than those trying to gain weight; however, results of a previous study (15) showed no significant difference in water intake among respondents trying to lose weight in the previous year than among those not trying to lose weight. Although there is a known significant negative association between energy intake and water consumption, evidence is less clear about the relationship between BMI and water intake. In our study, BMI and water intake levels were unrelated after models controlled for sociodemographics and health-related variables. There are at least 3 plausible explanations for this lack of association: 1) the self-reported BMI values of survey participants may be lower than the true values because survey respondents underestimated weight and overestimated height (27,28), thus decreasing our ability to detect an association; 2) the cross-sectional data did not allow us to assess whether prior behaviors of survey participants may have contributed to weight gain; and 3) our adjustments for factors closely associated with obesity, such as physical activity level or fruit and vegetable intake, may have masked the bivariate association we found between water intake and BMI.

### Study limitations

FAB data are cross-sectional and the survey results can show only an association between factors, not a causal relationship. The FAB sample was selected from a consumer opinions panel rather than the US population (due to declining response to random–digit-dial telephone surveys); this method is commonly used in other nutrition and health studies such as Styles (18). The response rate of 57% is similar to other random–digit-dial and consumer opinion mailed surveys; information on nonresponders was not available. To minimize bias, households from the larger consumer opinions panel pool selected for FAB were similar to the US population (by age, household income, geographic region, population density, and household size), and data were weighted using US Census estimates; however, these efforts do not guarantee lack of residual bias due to sample selection or nonresponse (18). FAB oversampled African Americans as part of the study design, but the sample size for “other” racial/ethnic groups was not sufficient for subgroup analyses. Dietary intake estimates were self-reported and may have been over- or underestimated and less accurate than data from surveys such as NHANES. Although the validity of the water intake question in FAB has not been determined, a recent study among adults found no significant difference between water intake that was self-reported on a questionnaire (on which the question was worded similarly to the question on the FAB) and water intake determined through 4-day food intake records (*r* = 0.7) (29). BMI data in FAB are determined on the basis of self-reported weight and height and subject to reporting bias; however, measured and self-reported BMI are highly correlated among adults (*r* > 0.9) (30). Finally, because the FAB data set did not include data about intake of calorically or artificially sweetened beverages, milk, or alcohol, we were unable to assess the relationship between intake of water and these beverages.

### Conclusion

Approximately 7% of respondents reported drinking no water daily, and nearly half reported drinking less than 4 cups per day. Low drinking water intake was associated with various unhealthful behaviors, including low levels of physical activity and low levels of fruit and vegetable intake. Models controlling for sociodemographics indicated that attitudes about eating and health, as well as food-related behaviors such as eating meals while watching television, were also related to low drinking water intake. Further studies of population samples with greater ability to assess differences in water intake by race/ethnicity subgroups (eg, Hispanics, Asians) are needed, as is research to learn where people consume drinking water, such as homes, worksites, or community venues. Our results suggest that low drinking water intake is common and is associated with known unhealthful behaviors. Clinical and public health practitioners aiming to help people drink more water should consider low water intake as part of a group of unhealthful behaviors and attitudes.
